# IqgC is a potent regulator of macropinocytosis in the presence of NF1 and its loading to macropinosomes is dependent on RasG

**DOI:** 10.1098/rsob.230372

**Published:** 2024-01-24

**Authors:** Darija Putar, Anja Čizmar, Xiaoting Chao, Marija Šimić, Marko Šoštar, Tamara Ćutić, Lucija Mijanović, Ana Smolko, Hui Tu, Pierre Cosson, Igor Weber, Huaqing Cai, Vedrana Filić

**Affiliations:** ^1^ Division of Molecular Biology, Ruđer Bošković Institute, Bijenička cesta 54, 10000 Zagreb, Croatia; ^2^ National Laboratory of Biomacromolecules, Institute of Biophysics, Chinese Academy of Sciences, 100101 Beijing, People's Republic of China; ^3^ College of Life Sciences, University of Chinese Academy of Sciences, 100049 Beijing, People's Republic of China; ^4^ Department of Cell Physiology and Metabolism, Faculty of Medicine, University of Geneva, Geneva, Switzerland

**Keywords:** IqgC, Ras, NF1, Rab5, macropinocytosis, *Dictyostelium*

## Abstract

RasG is a major regulator of macropinocytosis in *Dictyostelium discoideum*. Its activity is under the control of an IQGAP-related protein, IqgC, which acts as a RasG-specific GAP (GTPase activating protein). IqgC colocalizes with the active Ras at the macropinosome membrane during its formation and for some time after the cup closure. However, the loss of IqgC induces only a minor enhancement of fluid uptake in axenic cells that already lack another RasGAP, NF1. Here, we show that IqgC plays an important role in the regulation of macropinocytosis in the presence of NF1 by restricting the size of macropinosomes. We further provide evidence that interaction with RasG is indispensable for the recruitment of IqgC to forming macropinocytic cups. We also demonstrate that IqgC interacts with another small GTPase from the Ras superfamily, Rab5A, but is not a GAP for Rab5A. Since mammalian Rab5 plays a key role in early endosome maturation, we hypothesized that IqgC could be involved in macropinosome maturation via its interaction with Rab5A. Although an excessive amount of Rab5A reduces the RasGAP activity of IqgC *in vitro* and correlates with IqgC dissociation from endosomes *in vivo*, the physiological significance of the Rab5A–IqgC interaction remains elusive.

## Graphical abstract

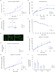

## 1. Introduction

Macropinocytosis is a clathrin- and dynamin-independent route of endocytosis used for nonselective, bulk uptake of extracellular fluid captured into large vesicles called macropinosomes [1,2]. In mammalian cells, macropinocytosis can be stimulated by growth factors or it can be constitutive, as is the case in some specialized immune cells, and many cancer cells [3,4]. The destiny of the internalized macropinosomes also depends on the cell type, reflecting different functions of macropinocytosis in various cellular contexts. In some cells, like EGF-treated A431, the digested content is almost completely recycled, while in others, like immature dendritic cells and macrophages, it undergoes lysosomal degradation [5]. Amoeba *Dictyostelium discoideum* also has degradative macropinosomes considering that macropinocytosis is a way of feeding for this unicellular organism. *Dictyostelium* and some mammalian macropinosomes arise from membrane protrusions that shape circular ruffles or macropinocytic cups, which close upon fusion of their distal membranes and finally pinch off from the plasma membrane [6–8]. Such extensive remodelling of the plasma membrane is achieved by Arp2/3 complex-nucleated dendritic F-actin network that pushes the membrane outwards [9]. At the same time, the protrusion is suppressed at the base of the cup where actin elongation factors, formins G and B in *Dictyostelium*, polymerize linear filaments that provide support [6,10,11]. Signals that guide cup formation and subsequent maturation of the nascent endosome are provided by the members of the Rho, Ras, Arf, and Rab families of small GTPases [6,12]. Simplified, in mammalian cells receptor tyrosine kinase (RTK) activated by its cognate growth factor activates class I phosphoinositide-3 kinase (PI3K) at the plasma membrane, which leads to rapid production of phosphatidylinositol (3,4,5)-trisphosphate (PI(3,4,5)P_3_). PI(3,4,5)P_3_ serves as a docking site for PH domain-containing proteins, among them GEFs and GAPs for Rac/Rho family GTPases that direct F-actin polymerization [9,13,14]. Although Ras is dispensable for macropinocytosis in mammalian cells [15], its activation in response to RTK activation contributes to PI3K activation and strongly enhances membrane ruffling and macropinocytosis [14,16]. Moreover, its role in macropinocytosis is gaining importance ever since macropinocytosis was recognized as a nutrient acquisition pathway that is intrinsically activated in tumour cells harbouring oncogenic Ras [17,18]. In this setting, Ras bypasses the activation of RTK and directly activates PI3K, independently of growth factors [19].

Similar to Ras-driven cancer cells, macropinocytosis in axenic *Dictyostelium* strains is constitutive and activated cell-autonomously [2,20]. However, unlike mammalian cells, in *Dictyostelium*, which contains a large repertoire of Ras family GTPases, RasG and RasS seem to be critical for macropinocytosis because cells depleted of these proteins show markedly decreased fluid uptake [21–23]. Consistently with their major role in macropinocytosis, these two Ras proteins are suggested to be the most potent activators of class I PI3Ks 1, 2, and 4, which are essential for the formation and closure of macropinocytic cups in *Dictyostelium* [22]. Furthermore, RasG recruits and activates formin G at the base of the cup thus additionally contributing to efficient macropinocytosis [10].

After the cup closure, actin is rapidly dismantled from the nascent macropinosome, PI(3,4,5)P_3_ is dephosphorylated to phosphatidylinositol (3,4)-bisphosphate (PI(3,4)P_2_), and plasma membrane components are recycled back to the cell surface [24]. Further maturation of degradative macropinosomes refers to fusion events; in early stages, fusions with other macropinosomes and early endosomes take place, and later with endolysosomes [25–27]. During early maturation events, Rab5 presence on nascent macropinosomes rapidly increases [27]. Rab5 activates early effectors, among them class III PI3K Vps34, which produces phosphatidylinositol 3-phosphate (PI(3)P) [28]. This leads to the recruitment of other effectors that cooperatively bind to both Rab5 and PI(3)P thus further driving macropinosome maturation [29].

Previously we have shown that *D. discoideum* IQGAP-related protein IqgC acts as a RasGAP, which specifically deactivates RasG on macropinosomes and phagosomes, thereby negatively regulating large-scale endocytosis [30]. Unexpectedly, the deletion of IqgC did not considerably increase the efficiency of macropinocytosis. Since the used parental strain is axenic and already has strongly upregulated fluid uptake, we hypothesized that deficiency of IqgC could induce a more prominent effect if introduced into the original genetic background of natural wild amoebas that inefficiently take up fluid. Therefore, we investigated the effects of *iqgC* deletion on both types of large-scale endocytosis in the non-axenic DdB strain and showed that IqgC plays an important role in the regulation of macropinocytosis in this genetic background. IqgC was also found to colocalize with an active Ras probe on the macropinocytic cup, but after internalization, the probe dissociated from the vesicle prior to IqgC [30]. This suggested RasG-independent functions of IqgC during early macropinosome maturation. We assumed that RasG recruits IqgC to macropinosomes, but that another interactor mediates its retention after the deactivation of Ras. Here, we explored requirements for IqgC recruitment to the forming macropinocytic cup and determined that interaction with RasG is indeed indispensable. In addition, we demonstrated that IqgC also interacts with the active form of the small GTPase Rab5A. This physical interaction did not promote the GTPase activity of Rab5A. Although Rab5A was initially considered as a candidate for mediating IqgC retention on the closed macropinosome, our results could not conclusively support its involvement in either retention or dissociation of IqgC from the macropinosome. Thus, the physiological significance of IqgC–Rab5A interaction remains unclear and awaits further elucidation.

## 2. Results

### 2.1. Deletion of *iqgC* in the NF1+ background significantly upregulates large-scale endocytosis

IqgC is a RasG-specific GTPase activating protein (GAP) that strongly accumulates at forming and nascent macropinosomes [30]. Nevertheless, the deletion of *iqgC* in axenic *D. discoideum* strain AX2 induced only a minor enhancement of fluid uptake that did not confer an advantage for cell growth in shaken suspension. In particular, there was no difference in the uptake 60 min after the addition of TRITC-dextran between wild-type and *iqgC* null cells, and only a minor difference was noticeable at later stages [30]. Whereas wild-type *D. discoideum* isolates preferentially feed on bacteria and have insufficient macropinocytic uptake to thrive in liquid media, axenic strains have been selected based on their ability to grow in the absence of bacterial food due to highly upregulated fluid uptake [2,31]. This constitutively high rate of macropinocytosis is achieved by the deletion of neurofibromin 1 (NF1), a RasGAP that strongly suppresses fluid and large particle uptake in wild amoebas [32]. We reasoned that the deletion of yet another RasGAP cannot appreciably advance the process that already operates at the almost maximal level.

**Figure 1.**
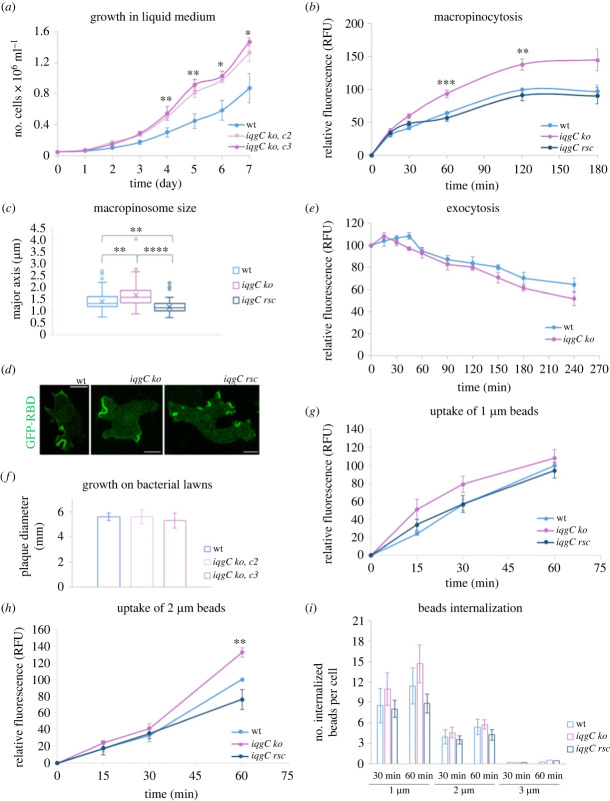
IqgC has a prominent role in the regulation of macropinocytosis in *D. discoideum* strain DdB. (*a*) *iqgC* null (*iqgC ko*) clones (*c2* and *c3*) have a significantly increased growth rate compared to parental (wt) cells when grown in a HL5 liquid nutrient medium supplemented with 10% FBS. (*b*) *iqgC* null cells show more efficient fluid phase uptake compared to parental cells and mutant cells whose phenotype was rescued by extrachromosomal expression of IqgC (*iqgC rsc*). (*c*) Cells deficient for IqgC form significantly larger macropinosomes than parental cells or cells with rescued IqgC expression, as judged by the size of the major macropinosome axis, *a*, measured upon macropinosome closure: *a*(wt) = 1.3 (1.2–1.6) µm, *n* = 80; *a*(*iqgC ko*) = 1.6 (1.4–1.9) µm, *n* = 77; *a*(*iqgC rsc*) = 1.1 (1.0–1.3) µm, *n* = 81 (median, interquartile range). (*d*) Representative images of wild-type, *iqgC* null cell, and *iqgC* null cell with rescued IqgC expression, expressing GFP-mRaf1_RBD. Only GFP channel is shown. (*e*) Wild-type and *iqgC* null cells have similar kinetics of the liquid medium exocytosis. (*f*) *iqgC* null clones and parental cells grow at a comparable rate on bacterial lawns. Plaque diameters were measured after 3 days. (*g*) *iqgC* null cells do not show significant increase in the uptake of beads of 1 µm in diameter compared to parental cells or cells with rescued IqgC expression. (*h*) *iqgC* null cells more efficiently accumulate 2 µm beads at 60 min compared to parental cells or cells with rescued IqgC expression. (*i*) Histogram showing the internalization of beads (1, 2, and 3 µm) at 30 and 60 min for parental, *iqgC* null, and rescued cells. Data are obtained from at least 3 independent experiments presented as mean ± SD in (*a*,*f*), or as mean ± SEM in (*b*,*e*,*g*–*i*). *p < 0.05; **p < 0.01; ***p < 0.001; ****p < 0.0001. Scale bars: 5 µm.

Therefore, we decided to investigate the effect of *iqgC* deletion in the NF1+ genetic background. We knocked out the *iqgC* gene in the DdB wild-type strain (electronic supplementary material, figure S1*a–c*) and compared the growth of two mutant clones under different conditions to show the consistency of phenotype (figure 1*a*,*f*). When cultivated in a liquid nutritive medium, *iqgC* null cells grew markedly better compared to parental cells (figure 1*a*). This notable improvement appears to be the consequence of considerably increased macropinocytosis efficiency of *iqgC* null cells (figure 1*b*), due to their significantly enlarged macropinosomes (figure 1*c*). Both the increased uptake and the size of macropinosomes could be reverted to wild-type values when IqgC was extrachromosomally expressed in *iqgC* null cells. Interestingly, the surface of rescued cells was often decorated with numerous small cups labelled with the active Ras probe (figure 1*d*). To rule out the possibility that defects in exocytosis contribute to the accumulation of fluorescently labelled medium in *iqgC* null cells, we also compared the kinetics of fluid phase exocytosis (figure 1*e*). As in the AX2 background [30], there was no difference in exocytosis between wild-type and *iqgC* null cells in the DdB background. Although *iqgC* null and parental cells grew at comparable rates on bacterial lawns (figure 1*f*), mutant cells showed slight, but statistically nonsignificant, increase in the uptake of small particles in shaken suspension (figure 1*g*), while the uptake of larger particles was enhanced only after 60 min (figure 1*h*). Internalization of 3 µm beads was almost completely suppressed in both cell lines (figure 1*i*). From these results, we conclude that IqgC has a more pronounced effect in the regulation of macropinocytosis in the NF1+ background than in the NF1− background. Moreover, its negative effect is additionally emphasized in mutant cells whose phenotype is rescued by IqgC overexpression (figure 1*b*,*c* and electronic supplementary material, figure S1*d*), thus further underlining the role of IqgC in restricting the size of macropinosomes.

### 2.2. RasG is required for the recruitment of IqgC to macropinocytic and phagocytic cups

**Figure 2.**
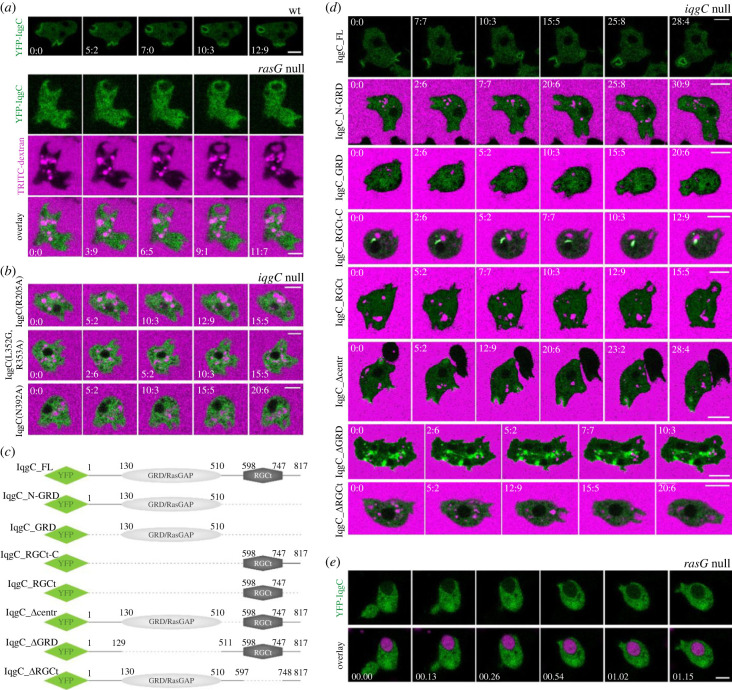
Recruitment of IqgC to endocytic cups is dependent on its interaction with RasG. (*a*) Unlike in wild-type cells (*upper panel*) YFP-IqgC signal is not sharply delineated on macropinocytic cups in *rasG* null cells (*lower panel*) during the uptake of the TRITC-dextran-labelled medium. (*b*) YFP-fused full-length IqgC mutants (R205A, L352G-R353A, N392A), which do not interact with RasG (electronic supplementary material, figure S2*a*), also do not localize to macropinosomes in *iqgC* null cells. (*c*) Schemes of YFP-fused full-length protein (IqgC_FL) and its truncated variants: IqgC_N-GRD, a protein containing N-terminal region and a RasGAP domain, also known as a GAP-related domain (GRD); IqgC_GRD, the RasGAP domain of IqgC; IqgC_RGCt-C, a protein containing a RasGAP_C-terminus (RGCt) domain and a C-terminal region; IqgC_RGCt, an RGCt domain of IqgC; IqgC_Δcentr, a protein lacking region between RasGAP and RGCt domains; IqgC_ΔGRD, a protein lacking the RasGAP domain; IqgC_ΔRGCt, a protein lacking the RGCt domain. Numbers denote amino acid positions in the protein. (*d*) Localizations of IqgC truncated variants in *iqgC* null cells during the uptake of the fluorescent medium were compared to the localization of the full-length IqgC. (*e*) YFP-IqgC does not localize to phagocytic cups in *rasG* null cells during the ingestion of TRITC-labelled yeast particles. Panels (*a*(*lower panel*),*b*,*d*,*e*) correspond to electronic supplementary material, movies S1–S12 and S14. In (*a*,*b*,*d*) time is given in seconds, and in (*e*) in the min:sec format. Scale bars: 5 µm.

We have previously demonstrated that IqgC colocalizes with active Ras during macropinosome formation and that active Ras probe dissociates from the internalized macropinosome before IqgC [30]. This finding suggests that IqgC may have RasG-independent functions during early macropinosome maturation and that RasG is not required for the retention of IqgC on the closed macropinosome. It has not been investigated, however, whether RasG is required for the recruitment of IqgC to forming macropinosomes. We, therefore, expressed fluorescently labelled IqgC in *rasG* null cells and found that, in the absence of RasG, IqgC does not localize to macropinosomes (figure 2*a* and electronic supplementary material, movie S1). This finding further confirms that IqgC specifically interacts with RasG during macropinosome formation. Next, we mutated several amino acid residues in the IqgC RasGAP domain known to be involved in the Ras–RasGAP interaction [33], and found that these RasG-nonbinding IqgC mutants are not recruited to forming macropinosomes (figure 2*b*; electronic supplementary material, figure S2*a* and movies S2–S4). From this, we conclude that interaction with RasG is indispensable for the recruitment of IqgC to macropinosomes.

To further evaluate which regions of IqgC are required for its recruitment to forming macropinocytic cups, we constructed several fluorescently labelled variants of IqgC (figure 2*c*) and expressed them in *iqgC* null cells. Only the protein lacking the region between RasGAP and RGCt domains (IqgC_Δcentr) had the same localization as the full-length IqgC (figure 2*d* and electronic supplementary material, movie S10). Interestingly, IqgC devoid of the RasGAP domain (IqgC_ΔGRD), which mediates IqgC binding to RasG (electronic supplementary material, figure S2*b*), showed localization all over the plasma membrane, except on macropinosomes (figure 2*d*; electronic supplementary material, figure S2*c* and movie S11). From these localizations, we infer that motifs responsible for the binding of IqgC to the plasma membrane are dispersed along the length of the polypeptide chain, but that the RasG-binding RasGAP domain guides IqgC specifically to the macropinosomal membrane.

IqgC also localizes to phagocytic cups, but with considerably lower abundance compared to macropinosomes [30]. Since the role of RasG in phagocytosis has not been systematically explored, we examined whether extrachromosomally expressed RasG localizes to phagocytic cups. We found that RasG localizes evenly along the cell membrane, including forming phagosome, from which it dissociates soon after the cup closure (electronic supplementary material, figure S2*d* and movie S13). Then we challenged *rasG* null cells expressing IqgC with fluorescently labelled yeast particles and observed that, as with macropinosomes, IqgC does not localize to phagosomes in the absence of RasG (figure 2*e* and electronic supplementary material, movie S14).

### 2.3. Interaction with RasG is a prerequisite for IqgC binding to the plasma membrane *in vivo*

**Figure 3.**
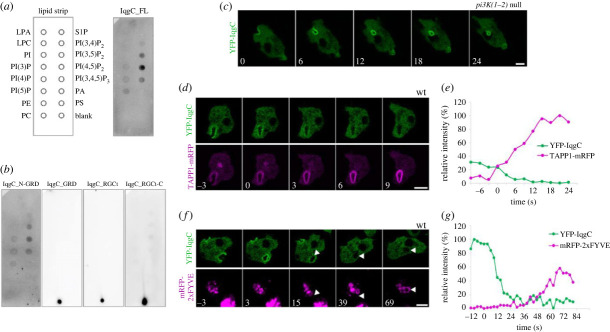
IqgC nonselectively interacts with all PIPs. Its recruitment to the forming macropinosome is not PI(3,4,5)P_3_-dependent but terminates during the conversion of PI(3,4,5)P_3_ to PI(3,4)P_2_. (*a*,*b*) Full-length IqgC (*a*) and IqgC_N-GRD (*b*) proteins expressed in *iqgC* null cells show the same binding affinities toward PIPs in a lipid dot blot assay. (*c*) YFP-IqgC localizes to the macropinosome in *pi3K(1-2)* null cells. (*d*) IqgC colocalizes with PI(3,4)P_2_ sensor TAPP1 during macropinosome formation, but with opposite kinetics. (*e*) Quantification of the relative signal intensity of YFP-IqgC and TAPP1-mRFP over time for the representative cell shown in (*d*). The closure of macropinosome occurs at time zero. (*f*) IqgC dissociates from the macropinosome before the recruitment of PI(3)P sensor 2xFYVE. (*g*) Quantification of the relative signal intensity of YFP-IqgC and mRFP-2xFYVE over time for the representative cell shown in (*f*). The closure of macropinosome occurs at time zero. Panels (*c*,*d*,*f*) correspond to electronic supplementary material, movies S15, S17 and S18. In (*c*,*d*,*f*) time is given in seconds. Scale bars: 5 µm. LPA, lysophosphatidic acid; LPC, lysophosphocholine; PI, phosphatidylinositol; PE, phosphatidylethanolamine; PC, phosphatidylcholine; S1P, sphingosine 1-phosphate; PA, phosphatidic acid; PS, phosphatidylserine.

Retention of IqgC on the internalized vesicle, after active Ras probe has dissociated, prompted us to search for other binding partners of IqgC. To look into the possible role of membrane phospholipids, we employed a lipid dot blot assay with cell lysates. Full-length wild-type IqgC protein expressed in *iqgC* null *D. discoideum* cells apparently interacts with all phosphoinositides (PIPs), with the highest affinity toward PI(4,5)P_2_ (figure 3*a*). Of note, IqgC purified from bacteria showed slightly different lipid specificity, with a strong binding to three PI-bisphosphates and PI(3)P (electronic supplementary material, figure S3*a*,*b*). Then we assessed the lipid binding of truncated IqgC variants expressed in *iqgC* null cells and showed that the RasGAP domain is not sufficient for binding to PIPs, but in conjunction with 129 N-terminal amino acid residues shows the same binding pattern as the full-length protein (figure 3*b*). Furthermore, none of the full-length RasG-nonbinding IqgC mutants retained the wild-type protein's capacity for binding to PIPs (electronic supplementary material, figure S3*c*). In addition, a lipid dot blot performed with the lysate of *rasG* null cells expressing full-length wild-type IqgC confirmed that interaction with RasG is required for the binding of IqgC to PIPs (electronic supplementary material, figure S3*d*). Taken together, these results suggest that the conserved interaction with RasG and the N-terminal region of IqgC are required for its binding to membrane phospholipids.

Next, we examined the localization of IqgC in *pi3K(1-2)* null cells and found that IqgC localizes to macropinosomes in cells heavily depleted of PI(3,4,5)P_3_ (figure 3*c* and electronic supplementary material, movie S15). Of note, the aforementioned abbreviation denotes the corresponding plasmanylinositol-phosphate because *Dictyostelium* mainly uses plasmanylinositols instead of phosphatidylinositols [34]. Given that *pi3K(1-2)* null cells still contain around 20% of wild-type PI(3,4,5)P_3_ [22], we also examined the localization of IqgC in *pten* null cells and observed the same localization as in wild-type cells (electronic supplementary material, movie S16). Namely, proteins whose membrane binding is PI(3,4,5)P_3_-dependent exhibit uniform plasma membrane localization in the absence of PI(3,4,5)P_3_ 3-phosphatase PTEN [35,36]. Altogether, these results suggest that binding to PI(3,4,5)P_3_ does not direct IqgC to macropinocytic cups, and are consistent with the exclusive role of RasG in the recruitment of IqgC to macropinosomes.

Finally, to determine the time point of IqgC dissociation from the closed macropinosome, we coexpressed IqgC with the PI(3,4)P_2_ sensor TAPP1 [37] and the PI(3)P sensor 2xFYVE [38]. We found IqgC present at the macropinosome during the accumulation of PI(3,4)P_2_, but it dissociated from the vesicle before PI(3,4)P_2_ reached a maximum (figure 3*d*,*e* and electronic supplementary material, movie S17). In line with that, IqgC vanishes long before PI(3)P buildup commences (figure 3*f*,*g* and electronic supplementary material, movie S18).

### 2.4. IqgC binds active Rab5A *in vitro* but does not promote its GTPase activity

**Figure 4.**
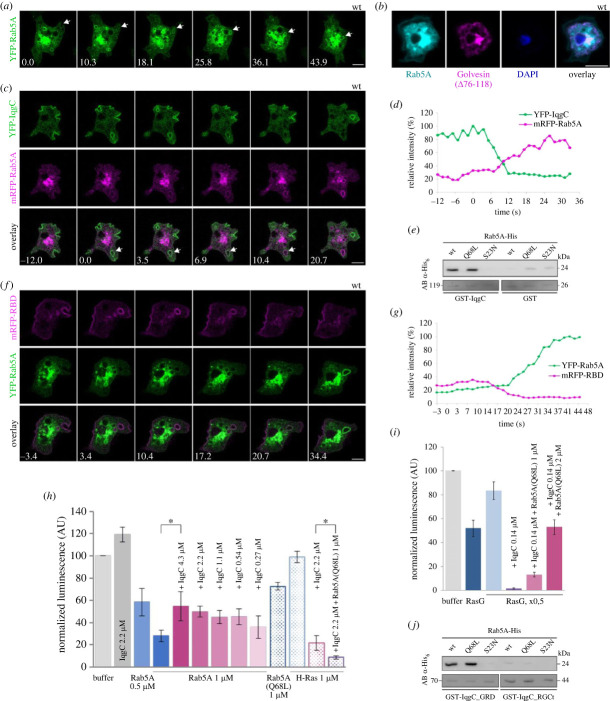
IqgC interacts directly with active Rab5A via its RasGAP domain. (*a*) YFP-Rab5A intensity gradually increases after the macropinosome closure. (*b*) YFP-Rab5A colocalizes with the probe for the core Golgi apparatus. (*c*) IqgC and Rab5A colocalize on the macropinosome but with opposite recruitment dynamics; after the cup closure, the IqgC signal decreases and it is completely gone before the Rab5A signal reaches its maximum. (*d*) Quantification of the normalized signal intensity of YFP-IqgC and mRFP-Rab5A over time for the representative cell shown in (*c*). The closure of macropinosome occurs at time zero. (*e*) Anti-His blot of GST-IqgC pull-down with purified His-tagged Rab5A variants shows that IqgC exclusively interacts with the active Rab5A. GST pull-down was performed as a negative control. (*f*) The active Ras probe and Rab5A colocalize on the macropinosome similarly to IqgC and Rab5A. (*g*) Quantification of the normalized signal intensity of YFP-Rab5A and mRFP-mRaf1_RBD over time for the representative cell shown in (*f*). The closure of macropinosome occurs at time zero. (*h,i*) Histograms showing results of GAP assays in which the GTPase activity reciprocally relates to the luminescence output (see Material and methods for details). (*h*) GAP assay with purified IqgC and Rab5A(wt) proteins (filled columns on the left side of the histogram) shows that intrinsic GTPase activity of 1 µM Rab5A does not increase in the presence of IqgC. Besides, increasing amounts of IqgC in the GTPase reaction produce more light corresponding to less GTP being hydrolysed. The dotted columns on the right side of the histogram show that Rab5A(Q68L) still possesses modest GTPase activity. Its addition to the GAP reaction does not inhibit the GAP activity of IqgC toward H-Ras (pink versus purple). (*i*) GAP assay with *D. discoideum* GST-RasG, immobilized on the glutathione-agarose, and fixed concentration of IqgC in the absence (purple) and presence of 1 µM or 2 µM Rab5A(Q68L) (pink shades). Blue columns represent intrinsic GTPase activities of GST-RasG on the slurry (one volume, dark blue; half the volume, light blue). (*j*) Anti-His blot of GST-IqgC_GRD and GST-IqgC_RGCt pull-downs with purified His-tagged Rab5A GTPases shows that active Rab5A binds to the RasGAP domain of IqgC. Panels (*a*,*c*,*f*) correspond to electronic supplementary material, movies S19–S21. In (*a*,*c*,*f*) time is given in seconds. Scale bars: 5 µm. The abbreviation AB in (*e*,*j*) signifies the staining with Amido Black. Data shown in (*h*,*i*) represent mean ± SD from at least 3 independent experiments. *p < 0.05.

After examining the interaction of IqgC with phospholipids, we reanalysed our mass spectrometry data of IqgC interacting proteins [30]. Another small GTPase from the Ras superfamily, Rab5A, attracted our attention as a promising candidate because Rab5 is a major regulator of early endosome maturation in mammalian cells [39]. First, we expressed YFP-Rab5A in AX2 cells and observed that it is weakly present at the cell membrane and during macropinosome formation, but its signal progressively increases after internalization and reaches a maximum during centripetal movement of endosomes towards perinuclear space and Golgi apparatus (figure 4*a*,*b* and electronic supplementary material, movie S19). On the contrary, IqgC is enriched at the forming macropinosome from the activation of the membrane patch up to the cup closure, and its signal starts to decrease only after internalization [30]. Thus, although IqgC and Rab5A exhibit opposite recruitment dynamics, they do colocalize on the macropinosome for the most part during macropinosome formation and until IqgC has dissociated from the vesicle (figure 4*c*,*d* and electronic supplementary material, movie S20). To examine whether IqgC and Rab5A interact directly, we performed GST-Rab5A pull-down assay with purified IqgC and showed that IqgC binds to Rab5A (electronic supplementary material, figure S4). Since IqgC bound considerably to GST as well, we reproduced the interaction in a GST-IqgC pull-down assay with purified His-tagged Rab5A, using wild-type (wt), constitutively active (Q68L), and constitutively inactive (S23N) Rab5A variants. IqgC strongly interacted with Rab5A(wt) and Rab5A(Q68L), but not with Rab5A(S23N) (figure 4*e*).

Given that Rab5A coexists with IqgC on the macropinosome, it is not surprising that it also coexists with active Ras (figure 4*f*,*g* and electronic supplementary material, movie S21). To explore the interplay between IqgC and the two GTPases it interacts with, first we evaluated whether IqgC could be a GAP for Rab5A, as well as for RasG. This would not be without precedence, since the RasGAP domain of mammalian p120^GAP^/RASA1 binds both H-Ras and Rab5 and stimulates the GTPase activity of both GTPases [40]. To that end, we performed a luminescence-based GAP assay with purified Rab5A(wt) and IqgC proteins. By measuring the GTP-hydrolytic activity of Rab5A in the absence and presence of IqgC, we learned that IqgC shows no GAP activity towards Rab5A (figure 4*h*). Moreover, IqgC reduced the intrinsic GTPase activity of Rab5A in a concentration-dependent manner, as judged by the increased luminescence output in the presence of IqgC. Of note, this result should be taken with caution because the increase in luminescence could be, at least partly, attributed to the intrinsic luminescence of the IqgC sample. Next, we evaluated whether Rab5A influences the IqgC GAP activity toward H-Ras and determined that Rab5A(Q68L) does not inhibit its GAP activity toward human H-Ras (figure 4*h*). We even observed a reduction in the luminescence output in the presence of an equimolar amount of Rab5A(Q68L), which might indicate a Rab5A-induced increase in IqgC GAP activity toward H-Ras. Nevertheless, this is probably a consequence of combined GTPase activities of IqgC-stimulated H-Ras and Rab5A(Q68L), the latter still detectable *in vitro* (figure 4*h*). Then we proceeded to examine whether Rab5A influences the IqgC GAP activity toward its endogenous cognate GTPase, *D. discoideum* RasG. We performed an on-the-column GAP assay with immobilized GST-tagged RasG as described previously [30], in the absence and presence of Rab5A(Q68L). In contrast to its effect on the IqgC GAP activity toward human H-Ras, we found that Rab5A inhibits its GAP activity toward RasG in a concentration-dependent manner (figure 4*i*), suggesting that Rab5A might interfere with RasG binding to IqgC. However, it has to be noted that a substantial molar excess of Rab5A was required for this inhibition, thus questioning its physiological importance. Next we examined whether Rab5A binds to the IqgC RasGAP domain similarly to RasG (electronic supplementary material, figure S2*b*). We performed a pull-down assay with affinity-purified RasGAP and RGCt domains and observed that Rab5A interacts with the RasGAP, and not the RGCt domain (figure 4*j*). As expected, the binding was Rab5A-GTP-specific suggesting that IqgC could be an effector of Rab5A.

### 2.5. Overexpression of Rab5A correlates with a premature dissociation of IqgC from the macropinosome

**Figure 5.**
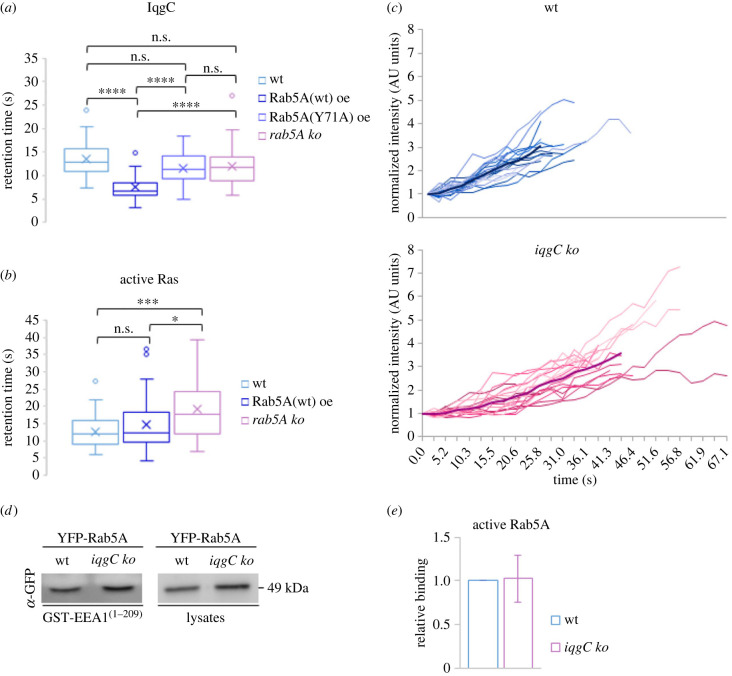
Overexpression of wild-type Rab5A correlates with a premature dissociation of IqgC from the macropinosome, while the loss of Rab5A does not affect the retention of IqgC but increases the lifetime of active Ras. (*a*) The retention times of IqgC on macropinosomes after internalization were determined in wild-type (wt), Rab5A(wt)-overexpressing (Rab5A(wt) oe), Rab5A(Y71A)-overexpressing (Rab5A(Y71A) oe), and *rab5A* null (*rab5A ko*) cells. The retention times were: *t*(IqgC/wt) = 13.0 (10.9–15.5) s, *n* = 34; *t*(IqgC/Rab5A(wt) oe) = 6.8 (5.8–8.3) s, *n* = 33; *t*(IqgC/Rab5A(Y71A) oe) = 11.4 (9.1–13.8) s, *n* = 36; *t*(IqgC/*rab5A ko*) = 11.7 (8.9–14.1) s, *n* = 67 (median, interquartile range). (*b*) The retention times of active Ras probe on macropinosomes after internalization were determined in wild-type, Rab5A(wt)-overexpressing, and *rab5A* null cells. The retention times were: *t*(Ras/wt) = 12.0 (9.2–15.9) s, *n* = 37; *t*(Ras/Rab5A(wt) oe) = 12.4 (9.7–18.3) s, *n* = 60; *t*(Ras/*rab5A ko*) = 17.7 (12.3–24.0) s, *n* = 40 (median, interquartile range). (*c*) The YFP-Rab5A intensities on early macropinosomes were followed upon their closure in wild-type (wt) and *iqgC* null (*iqgC ko*) cells. Thin lines depict YFP intensities on individual macropinosomes over time. The thick line on each graph represents the averaged intensities for each time point and is drawn up to the time point calculated as an average of all individual lifetimes. (*d*) Anti-GFP blot of GST-EEA1^(1–209)^ pull-down with lysates of wild-type (wt) and *iqgC* null (*iqgC ko*) cells expressing YFP-Rab5A. A blot with input lysates was used as a reference for densitometry. (*e*) Densitometric analysis of the relative binding of active YFP-Rab5A to EEA1^(1–209)^ in wild-type and *iqgC* null cells shows that there is no significant difference between the two. All data are gathered from at least 3 independent experiments. Data in (*e*) represent mean ± SD. *p < 0.05; ***p < 0.001; ****p < 0.0001; n.s., not significant.

Previously we have observed that the probe for active Ras dissociates from the internalized macropinosome before IqgC. However, when we independently analysed retention times of IqgC and active Ras probe in wild-type AX2 cells, we observed modestly prolonged retention of IqgC that was not statistically significant: *t*(IqgC/wt) = 13.0 (10.9–15.5) s, *n* = 34; *t*(Ras/wt) = 12.0 (9.2–15.9) s, *n* = 37 (median, interquartile range) (figure 5*a*,*b*, compare data for wt). Interestingly, while observing cells coexpressing exogenous IqgC and Rab5A, we noticed that IqgC falls off of the macropinosome sooner than in cells with an endogenous level of Rab5A. Indeed, the quantification revealed a significant reduction of the IqgC retention time in Rab5A-overexpressing cells (figure 5*a*). This suggested that Rab5A might promote dissociation of IqgC from the macropinosome. To investigate this hypothesis, we generated *rab5A* null cells (electronic supplementary material, figure S1*e*,*f*). Contrary to our expectations, the retention of IqgC on macropinosomes devoid of Rab5A was similar to wild-type cells (figure 5*a*). We additionaly examined the retention time of IqgC in cells expressing a mutant Rab5A, which has a reduced binding affinity for IqgC. To achieve this, we mutated amino acid residues in *D. discoideum* Rab5A that correspond to residues in mammalian Rab5, known to be involved in binding of different effector proteins [41–43]. Our aim was to find a Rab5A mutant that localizes to macropinosomes (electronic supplementary material, figure S5*a*) but has reduced affinity for IqgC. We identified the Rab5A(Y71A) mutant as a suitable candidate, because it had a satisfactory expression level and substantially reduced binding to IqgC compared to the wild-type Rab5A (electronic supplementary material, figure S5*b*). When IqgC was coexpressed with Rab5A(Y71A), the retention time of IqgC on the macropinosomes increased toward the value in wild-type cells, while being significantly different from the retention time in cells overexpressing Rab5A(wt) (figure 5*a*). Interestingly, overexpression of Rab5A did not significantly affect the retention time of the probe for active Ras (figure 5*b*). However, in the absence of Rab5A, the active Ras probe persisted on the macropinosome significantly longer (figure 5*b*).

Next, we wanted to investigate whether IqgC influences the dynamics of Rab5A. We analysed the intensity of YFP-Rab5A on nascent macropinosomes in wild-type and *iqgC* null cells. In the absence of IqgC, we only noticed a somewhat slower accumulation of Rab5A on the closed macropinosome (figure 5*c*). Finally, since IqgC binds the active Rab5A, it could modulate the Rab5A GTPase cycle. Therefore, we compared the levels of active Rab5A in wild-type and *iqgC* null cells using GST-EEA1^(1–209)^ as bait (figure 5*d*). However, there was no difference in the level of total active Rab5A in the absence of IqgC (figure 5*e*).

## 3. Discussion

### 3.1. IqgC is an important regulator of macropinocytosis in wild-type *D. discoideum* strain

IqgC is an IQGAP-related protein that has conserved RasGAP activity and negatively regulates large-scale endocytosis by inactivating small GTPase RasG on macropinosomes and phagosomes [30]. Having in mind the major role that RasG plays in the regulation of macropinocytosis in *D. discoideum*, it was somewhat surprising that the deletion of IqgC, its negative regulator in this process, induced only a modest upregulation of the fluid uptake in the AX2 background, manifested by slightly increased macropinosome size. On the other hand, it is known that axenic *D. discoideum* strains, such as AX2, that are widely used in laboratories, have significantly upregulated both types of large-scale endocytosis when compared to genuine wild-type strains. It was demonstrated that the deletion of the *axeB* gene, encoding NF1 in wild-type amoebae, brings about a major contribution to the axenic phenotype [32]. NF1 is a RasGAP that restricts the size of both macropinosomes and phagosomes in wild amoebas, and its absence together with other mutations, grants cells a major advantage for axenic growth in liquid media and for phagocytosis of large particles [32,44]. We, therefore, hypothesized that the elimination of another negative regulator of large-scale endocytosis cannot significantly upregulate the process that has already been genetically improved in axenic cells. That is why we examined the effect of *iqgC* deletion in DdB, the parent natural strain from which AX2 is derived [45]. Indeed, when *iqgC* was deleted in this original genetic background, we observed a significant improvement in axenic growth due to an increased macropinocytosis efficiency (figure 1*a*,*b*). We also observed a twofold higher increase in the major macropinosome axis upon closure, compared to the increase observed in the *iqgC* mutant generated in the AX2 background, which translates into an even higher increase in the macropinosome volume. *iqgC* null cells also exhibited a slight increase in the uptake of 1 and 2 µm beads (figure 1*g*,*h*), while ingestion of larger particles (3 µm) was precluded for mutant as well as for parental DdB cells (figure 1*i*). This is in contrast to the *axeB* null mutant in DdB background, which ingested bacterium-sized beads (1 and 1.8 µm) with the same efficiency as parental cells, but showed strikingly increased capacity for uptake of yeast-sized particles, while parental cells also could not significantly ingest 3 µm beads [32]. These results imply that, although both NF1 and IqgC negatively regulate fluid and particle uptake in wild amoebas, their roles are not redundant. These functional differences could be imposed by different affinities toward Ras GTPases, with IqgC being specific for RasG, whereas NF1 presumably acts on multiple Ras proteins involved in large-scale endocytosis, including RasG [44]. In addition, IqgC lacks a PH-like domain, and its recruitment to macropinosomes and phagosomes does not require PI(3,4,5)P_3_ but is dependent entirely on RasG (see below).

There are two other RasGAPs, RasGAP2/C2GAP2 and RGBARG, involved in large-scale endocytosis in *D. discoideum* [44,46,47]. C2GAP2, encoded by *c2gapB* gene, localizes to macropinocytic and phagocytic cups and nascent macropinosomes and phagosomes [46,47]. However, the observed effects on macropinocytic uptake upon *c2gapB* deletion are not in agreement. Li *et al*. have shown that, although having excessive patches of active Ras, *c2gapB* null cells show decreased fluid uptake [46]. Xu *et al*. examined macroendocytic uptake of *c2gapB* null cells and identified C2GAP2 as a negative regulator of both macropinocytosis and phagocytosis, and consequently of the cell growth [47]. RGBARG is a RasGAP/RapGAP enriched at the protrusive rim of both macropinocytic and phagocytic cups [44]. *RGBARG* null cells have increased Ras/PI(3,4,5)P_3_ patches but produce smaller macropinosomes and have significantly reduced fluid-phase uptake in the AX2 background. This phenotype is most likely the combination of increased Ras activity and reduced Rac activity because RGBARG also harbours a RhoGEF domain. Interestingly, *RGBARG* null cells, similar to *iqgC* null cells in AX2 background, have significantly increased phagocytosis of large particles.

In recent years we have gathered plenty of data on how multiple RasGAPs regulate the size and shape of large endocytic cups. However, the emerging picture is still blurred by seemingly different effects exerted by the same protein on macropinocytosis and phagocytosis in NF1+ and NF1− genetic backgrounds.

### 3.2. RasG recruits IqgC to the forming macropinosomes

Previously, we have observed that IqgC transiently translocates to the plasma membrane in response to a uniform stimulation with folic acid [30], in a similar manner to active Ras, PI3K and PI(3,4,5)P_3_ probes [48]. Macropinocytic cups in *Dictyostelium* emerge from membrane patches highly enriched in active Ras and PI(3,4,5)P_3_ [49], and IqgC fully coincides with active Ras. Here, we investigated the requirements for IqgC recruitment to membrane domains primed for macropinocytosis. By examining the localization of IqgC during the uptake of the fluorescently labelled liquid medium in *rasG* null background (figure 2*a*), or of RasG-nonbinding IqgC mutants in *iqgC* null background (figure 2*b*), we demonstrated that IqgC does not localize to forming macropinosomes when its interaction with RasG is precluded. In addition, truncated IqgC lacking its RasG-binding RasGAP domain (IqgC_ΔGRD) localizes to the plasma membrane but is excluded from the incipient macropinocytic cups (figure 2*d*), further confirming that RasG recruits IqgC to the forming macropinosome. Of note, IqgC_ΔGRD also has strong signal from cytosolic structures, which we suspect are protein aggregates resulting from overexpression of this truncated variant of IqgC.

Interestingly, mutation of the arginine finger in the RasGAP domain of IqgC (R205A) abrogates its interaction with RasG (electronic supplementary material, figure S2*a*) and its recruitment to the macropinocytic cup (figure 2*b*). This is in contrast to the accepted notion that mutation of catalytically active arginine residue dramatically reduces GAP activity of RasGAPs, but does not affect their binding to Ras [50,51]. It should be mentioned, however, that severely impaired association with Ras was also reported [52]. In comparison to *Dictyostelium* RasGAPs, catalytically inactive, arginine finger mutants of C2GAP2 and C2GAP1 bind Ras, and C2GAP1(R616A) translocates to the membrane in response to cAMP stimulation similar to the wild-type protein [47,53]. Furthermore, both *Dictyostelium* NF1 and RGBARG harbouring mutated arginine finger, although not able to rescue the RasGAP-dependent phenotype of mutant cells, localized to macropinocytic cups like wild-type proteins [32,44]. In general, RasGAPs are modular proteins that contain, besides the Ras-interacting RasGAP domain, other domains responsible for phospholipid- and protein-binding that mediate their translocation to the membrane [54]. However, unlike mammalian RasGAPs and *Dictyostelium* NF1, RGBARG and C2GAPs, IqgC contains no obvious lipid-binding motif. Localization experiments with truncated IqgC variants suggest that amino acid residues important for its recruitment to the macropinosome do not reside in one domain, but are most likely distributed in different regions (figure 2*d*). Nevertheless, the functional RasGAP domain seems to be crucial for the guidance to the macropinosomal membrane because, peculiarly, the full-length protein lacking this domain becomes mislocalized along the entire plasma membrane, except for the macropinosomes. This might reflect the observed strongest binding of IqgC to PI(4,5)P_2_ (figure 3*a*) since the localization of IqgC_ΔGRD is comparable to the localization described for PTEN [22].

We also demonstrated that recruitment of IqgC to macropinocytic cups is not PI(3,4,5)P_3_-dependent (figure 3*c* and electronic supplementary material, video S15), although the full-length protein binds to PI(3,4,5)P_3_ in a lipid dot blot (figure 3*a*). Nevertheless, in this *in vitro* assay IqgC shows nonselective binding to all PIPs, with the highest affinity for PI(4,5)P_2_ (figure 3*a*). We did not determine the minimal motif required for the binding, but 129 N-terminal amino acids in tandem with a functional RasGAP domain seem to be required for the interaction with PIPs (figure 3*b* and electronic supplementary material, figure S3*c*). Finally, IqgC expressed in cells deficient for RasG did not interact with any of the PIPs (electronic supplementary material, figure S3*d*). Based on these results, we conclude that RasG recruits its own GAP to the forming macropinosomes, while interactions with PIPs presumably stabilize the association of IqgC with the membrane.

### 3.3. IqgC interacts with Rab5A, another small GTPase present on the macropinosome

Seeking to characterize other signalling pathways that involve IqgC, in addition to its role in the regulation of RasG activity, we identified Rab5A as its direct interactor (figure 4*e* and electronic supplementary material, figure S4). While little is known about *Dictyostelium* Rab5A, mammalian Rab5 is a well-established early endosome marker that plays a central role in the maturation of early to late endosomes [55]. The delivery of Rab5 to the membrane is guided by its specific GEF, which activates it and ensures that it is recruited to the correct target membrane [56]. In order for Rab5a to be properly directed to the early endosomes, it is essential that it interacts with its cognate GEF, Rabex-5, which is present on the early endosome membrane [57]. Recently, it has been discovered that the activation and recruitment of Rab5a to macropinosomes specifically require the Rab5 GEF ALDS2 [58]. Once at the membrane Rab5, via its numerous effectors, mediates multiple functions on early endosomes. Its early effector Rabaptin-5 binds Rabex-5, releases it from autoinhibition, and thus participates in the feedback loop by which Rab5 positively regulates its own activity [59,60]. By activating Vps34 and binding tethering molecules, such as EEA1, Rab5 promotes the fusion of primary endocytic vesicles, including macropinosomes, with early endosomes, and homotypic fusion of early endosomes [27,28,61–63]. Rab5 also controls the conversion of Rab5-positive early endosomes to Rab7-positive late endosomes by orchestrating its own deactivation [39]. It binds a Rab7 GEF, the Mon1-Ccz1 complex that activates Rab7 and displaces Rabex-5 from the membrane [64,65]. *D. discoideum* Rab5A has not been investigated thus far, but it was identified as a part of the LCV (*Legionella-containing vacuole*), a special type of phagocytic vesicle that forms during the infection with bacteria *Legionella pneumophila* [66]. So far, no obvious orthologues of mammalian Rab5 GEFs or effectors have been identified in *D. discoideum*. A recent study described TbcrA as a Rab5 GAP that, in complex with PripA, inactivates Rab5 while promoting Rab5-to-Rab7 macropinosome maturation [67].

We show that IqgC colocalizes with Rab5A on the nascent macropinosome, although their recruitment dynamics are opposite (figure 4*c*,*d*). Specifically, the IqgC signal is waning at the onset of increased Rab5A accumulation. In addition, coexpressions with probes specific for PI(3,4)P_2_ and PI(3)P confirm that IqgC dissociates from the nascent macropinosome at an early stage after internalization (figure 3*d*,*f*), thus placing IqgC in the early phase of macropinosome maturation, before Vps34 activation. Using pull-down and GAP assay with purified proteins, we demonstrated that IqgC interacts with active Rab5A, but it does not stimulate its GTPase activity (figure 4*e*,*h*). Moreover, IqgC seems to inhibit the intrinsic GTPase activity of Rab5A in a concentration-dependent manner. As already mentioned, we suspect that part of the luminescence increase could be due to the intrinsic luminescence of the IqgC sample (figure 4*h*, compare to the buffer alone). Nevertheless, it is conceivable that IqgC inhibits the GTPase activity of Rab5A by sterically preventing GTP hydrolysis. Human p120^GAP^/RASA1 is a RasGAP that interacts with active forms of H-Ras, Rap1 and Rab5 [40,68,69]. Rap1-GTP binds with high affinity to p120^GAP^/RASA1 and, although this interaction does not stimulate Rap1 GTPase activity, it competitively inhibits the p120^GAP^/RASA1-stimulated GTPase activity of Ras [69]. Interestingly, IqgC also binds *Dictyostelium* RapA and does not stimulate its GTPase activity (Mijanović *et al*., in preparation). RasGAP domain of p120^GAP^/RASA1 also binds active Rab5 with high affinity, and again a competition between Rab and Ras was observed. However, unlike with Rap1, this interaction stimulates the GTP hydrolysis on Rab5 [40]. Although we observed a significant decrease in the RasGAP activity of IqgC only in the presence of a considerable excess of Rab5A *in vitro*, our results indicate a possible competition between RasG and Rab5A for binding to IqgC (figure 4*i*).

Besides being the master regulator of early endocytic pathways, inhibition of macropinocytosis induced by overexpression of dominant negative Rab5 or by silencing of the three Rab5 isoforms in mammalian cells suggested that Rab5 plays a vital role during macropinosome formation [70,71]. Correspondingly, in addition to localizing to early endosomes, including macropinosomes, phagosomes, and clathrin-coated vesicles, Rab5 also localizes to the plasma membrane [27,72–74]. More specifically, Rab5 was reported to accumulate in plasma membrane ruffles just after ruffle closure, i.e. to open macropinocytic cups, and this accumulation precedes the loss of PI(4,5)P_2_ [71,75–77]. Rab5 was implicated in the signalling pathway leading to actin remodelling and circular ruffle formation downstream from activated RTK [78]. By contrast, a recent study showed that Rab5 is dispensable for membrane ruffling and, instead, plays an essential role downstream of membrane ruffling [71]. The authors showed that active Rab5 reaches the membrane in the form of Rab5-positive vesicles, that fuse with the circular ruffle membrane and brings along the 5-phosphatase activity, thus contributing to the depletion of PI(4,5)P_2_ required for macropinosome sealing.

*D. discoideum* Rab5A is also present at the plasma membrane, macropinosomes, and phagosomes (figure 4*a*,*b*) [67] but, contrary to mammalian cells, a PI(4,5)P_2_ spike around the ruffle closure has not been observed in this organism [79]. As a technical point, we used Rab5A expressed from an extrachromosomal vector. This resulted in a high overexpression of Rab5A that made it difficult to monitor low signal at the membrane and very strong signal in the perinuclear space at the same time due to the insufficient dynamic range of microscope detectors. In comparison, Tu *et al*. [67] used AX2 cells in which GFP-Rab5A was expressed from an expression cassette integrated as a single copy in the genome. This resulted in lower levels of overexpression that enabled better visualization of the plasma membrane localization of Rab5A.

We initially hypothesized that Rab5A could be the binding partner of IqgC that mediates its retention on the closed macropinosome after RasG has been deactivated. However, quantification of retention times of IqgC and the probe for active Ras on the internalized macropinosomes revealed minimal difference (figure 5*a*,*b*). Moreover, a shorter retention of IqgC in Rab5A-overexpressing cells suggested the opposite, i.e. that Rab5A promotes dissociation, not retention, of IqgC from the vesicle (figure 5*a*). Nevertheless, our efforts to substantiate the Rab5A-dependent dissociation of IqgC were inconclusive. While overexpression of wild-type Rab5A significantly shortened the presence of IqgC after the cup closure, we failed to observe reversed effect in the absence of Rab5A. On the other hand, overexpression of the mutant Rab5A, which localizes to the macropinosome but has reduced binding to IqgC, reverted the retention time of IqgC toward the value in wild-type cells. Therefore, additional approaches are needed to clarify whether the observed effect is indeed the consequence of Rab5A-IqgC interaction or merely perturbation in the system caused by protein overexpression.

One would expect that premature dissociation of IqgC under the condition of Rab5A(wt) overexpression would be accompanied by an increased lifetime of active Ras probe, due to insufficient inactivation of RasG. However, this was not the case. On the contrary, the lifetime of active Ras was significantly prolonged on macropinosomes in the absence of Rab5A (figure 5*b*). In mammalian cells, active Ras recruits and activates Rab5 on the macropinosomes by interacting with Rab5 GEF RIN1 [27,75,80]. Ras activation peaks almost concurrently with the maximal localization of Rab5, and Ras leaves Rab5-positive macropinosome, possibly via a fast recycling route, before fusion with other endocytic vesicles [75,77]. Analogously, in *Dictyostelium* cells deficient for Rab5A, both homotypic and heterotypic fusions of macropinosomes with other vesicles and/or early endosomes could be diminished, leading to prolonged retention of Ras on macropinosomes delayed in maturation.

Finally, we analysed the impact of *iqgC* deletion on Rab5A dynamics on the internalized macropinosome and the Rab5A activity. Our results show that IqgC does not affect the level of active Rab5A (figure 5*e*). A small delay in the initial increase of YFP-Rab5A signal after macropinosome closure in the absence of IqgC (figure 5*c*) is not sufficient to propose the influence of IqgC on Rab5A dynamics.

In summary, we have shown that interaction between IqgC and RasG is indispensable for the recruitment of IqgC to the forming macropinosome. The other identified IqgC interactor, Rab5A, is weakly present on the forming macropinocytic cup and starts to accumulate on the membrane of the primary endocytic vesicle after the cup closure. It is tempting to speculate that RasG and Rab5A compete for binding to IqgC, and that the rising concentration of Rab5A on the macropinosome increases its binding to IqgC at the expanse of RasG. Thus, disruption of its interaction with RasG would lead to the dissociation of IqgC from the vesicle. However, if that is the case, this role of Rab5A must be shared with or compensated for by another interactor of IqgC, because in the absence of Rab5A, IqgC still dissociates from the vesicle in a timely manner (figure 5*a*). Thus, further experiments are needed to clarify the role of IqgC interaction with Rab5A.

## 4. Material and methods

### 4.1. Plasmids

Homologous recombination was used to generate *iqgC* null cells in the DdB background and *rab5A* null cells in the AX2 background. To generate *iqgC* null cells, the 5′-region of homology was NgoMIV/HindIII cloned, and the 3′-region of homology of the *iqgC* gene was BglII/SpeI cloned in the pDM1081 vector, which contains hygromycin resistance cassette [81]. For the production of *rab5A* null mutant, a construct was made with SalI/HindIII cloned 5′-region of homology, and PstI/BamHI cloned 3′-region of homology of the *rab5A* gene in pLPBLP vector, which contains blasticidin resistance cassette [82]. To rescue the phenotype of *iqgC* null cells, a coding DNA sequence (CDS) of IqgC was BglII/SpeI cloned into the pDM1208 plasmid [81]. To compare the macropinosome sizes between DdB wild-type and *iqgC* null cells, and to determine the retention time of the active Ras on macropinosomes, a pDM1207 vector [81] with BglII/SpeI cloned mRaf1_RBD was used. To determine the macropinosome size in *iqgC* null cells with rescued IqgC expression, the expression cassette containing GFP-mRaf1_RBD was NgoMIV cut from the pDM1019_mRaf1_RBD shuttle vector and ligated into the NgoMIV site of pDM1208_IqgC.

For the expression of truncated variants of IqgC in *D. discoideum* cells, CDSs of IqgC_N-GRD (amino acid residues 1–510), IqgC_GRD (130–510), IqgC_RGCt (598–747), and IqgC_RGCt-C (598–817) were PCR-amplified, and BglII/SpeI cloned into the pDM304_N-YFP vector [10]. The same plasmid was used to create YFP-labelled IqgC_Δcentr (Δ511–597), IqgC_ΔGRD (Δ130–510), and IqgC_ΔRGCt (Δ598–747) truncated variants inserted by ligation-independent cloning (In-Fusion® HD Cloning Kit, Takara Bio). For the expression of full-length mutant variants of IqgC (R205A; L352G, R353A; N392A), site-directed mutagenesis was performed on the pDM304_YFP-IqgC vector [30]. To express fluorescently labelled Rab5A in *D. discoideum* cells, Rab5A CDS was amplified by PCR from the cDNA of AX2 cells and BamHI/SpeI cloned into the pDM304_N-YFP. The obtained pDM304_YFP-Rab5A plasmid was further used to coexpress YFP-Rab5A and mRFP-mRaf1_RBD; the expression cassette containing mRaf1_RBD was NgoMIV cut from the pDM328_mRaf1_RBD shuttle vector and ligated into the NgoMIV site of pDM304_YFP-Rab5A. To coexpress mRFP-Rab5A with golvesin(Δ76–118)-GFP or YFP-mRaf1_RBD, the mRFP-Rab5A expression cassette was NgoMIV cut from pDM328_Rab5A shuttle vector and cloned into the pDM323_golvesin(Δ76–118) [83] (a kind gift from Dr Hubert Hilbi) or pDM304_YFP-mRaf1_RBD [30] vector, respectively. For simultaneous expression of YFP-IqgC with mRFP-Rab5A, the expression cassette containing Rab5A was NgoMIV cut from corresponding pDM328 shuttle vector and ligated into the pDM304_YFP-IqgC vector. For the expression of full-length mutant variants of Rab5A (S23N; F46A; W63A; R70E; Y71A; M77S; R80E), site-directed mutagenesis was performed on the pDM304_YFP-Rab5A vector. For coexpression of YFP-IqgC and mRFP-Rab5A(Y71A), site-directed mutagenesis was performed on the pDM304_YFP-IqgC_mRFP-Rab5A vector. For coexpression of YFP-IqgC with TAPP1-mRFP, pDM304_YFP-IqgC and previously constructed pDM451_TAPP1 [67] were co-transfected into cells. 2xFYVE was cut from previously constructed pDM317_2xFYVE [67] and cloned into pDM449, and the resulting construct was then co-transfected with pDM304_YFP-IqgC for co-expression of mRFP-2xFYVE and YFP-IqgC. Previously constructed pDM304_YFP-RasG(wt) was used for the expression of fluorescently labelled RasG [10].

In yeast two-hybrid assay derivatives of pGBKT7 and pGADT7 vectors were used (Matchmaker™ GAL4 Two-Hybrid System 3, Clontech). pGBKT7_RasG(Q61L) and pGADT7_IqgC were constructed previously [30]. To produce pGADT7 encoding full-length mutant variants of IqgC (R205A; L352G, R353A; N392A), site-directed mutagenesis on pGADT7_IqgC was performed. To produce pGADT7 vector encoding RasGAP (i.e. GRD) or RGCt domain of IqgC, respective CDSs were amplified by PCR and BamHI/XhoI cloned into the pGADT7 vector.

For the production of C-terminally His-tagged Rab5A variants used in pull-down and GAP assays, a CDS of Rab5A(wt), without three C-terminal codons, was amplified by PCR and NcoI/HindIII ligated into the pET28b vector. Site-directed mutagenesis on pET28b_Rab5A(wt) was performed to obtain pET28b_Rab5A(Q68L) and pET28b_Rab5A(S23N) vectors. For affinity purifications of GST-Rab5A(wt), GST-IqgC_GRD and GST-IqgC_RGCt used in pull-down assays, the corresponding CDSs were PCR-amplified and BamHI/SalI ligated into the pGEX-6P-1 vector. For the purification of GST-IqgC used to pull-down mutant Rab5A proteins, previously constructed pGEX-6P-1_IqgC vector was used [30]. For the purification of GST-RasG used in the GAP assay, the previously constructed pGEX-6P-1_RasG(wt) vector was used [30]. For purification of GST-EEA1^(1–209)^ (amino acids 1–209 of human early-endosomal antigen 1) used to pull-down of active YFP-Rab5A, previously constructed pGEX-4T-1_EEA1^(1–209)^ vector was used [67]. Oligonucleotides used in this work are listed in electronic supplementary material, table S1.

### 4.2. Cell culture

For the generation of *iqgC* null cells in non-axenic background, *D. discoideum* DdB strain was used (kind gift from Dr Robert R. Kay). Cells were cultivated at 22°C in cell culture dishes in SorMC buffer (15 mM KH_2_PO_4_, 2 mM Na_2_HPO_4_, 50 µM MgCl_2_, 50 µM CaCl_2_, pH 6.0) in the presence of *Klebsiella aerogenes* diluted to OD_600_ = 2, or on *K. aerogenes* lawns on SM agar plates (10 g l^−1^ peptone, 10 g l^−1^ glucose, 1 g l^−1^ yeast extract, 1 g l^−1^ MgSO_4_·7H_2_O, 2.2 g l^−1^ KH_2_PO_4_, 1 g l^−1^ K_2_HPO_4_, 15 g l^−1^ agar), as described in [81]. To prepare the *K. aerogenes* suspension, bacterial cells were grown overnight in SM liquid medium at 37°C, harvested by centrifugation, washed in SorMC buffer, and diluted to a density OD_600_ = 100 in SorMC buffer. For electroporation, vegetative cells were harvested from *K. aerogenes* bacterial lawn, resuspended, and washed once in cold H40 buffer (40 mM HEPES, 1 mM MgCl_2_, pH 7.0). 3.5 × 10^6^ cells were electroporated with 1 µg of plasmid DNA under conditions of square wave protocol with two pulses of 350 V and 8 ms duration separated by a 1-second interval. Cells transfected with extrachromosomal vectors were maintained in the presence of 10 µg ml^−1^ G418 (Gibco). To examine the growth in a liquid medium, DdB wild-type and *iqgC* null cells were washed of bacteria with SorMC buffer. 5 × 10^4^ cells were transferred to each well of 24-well culture plates in triplicate in HL5 medium without glucose (Formedium) supplemented with 18 g l^−1^ of maltose (Sigma) and 10% fetal bovine serum (FBS, Gibco). Growth was assessed for seven consecutive days using a cell counter (CellDrop, DeNovix). To examine the growth on bacterial lawns, DdB wild-type and *iqgC* null cells were diluted and cultivated with *K. aerogenes* suspension on SM agar plates. The diameters of plaques were measured after 3 days.

Axenic strain AX2 was used as the wild-type reference strain for all experiments with axenic cells and for the generation of *rab5A* null cells. *rasG* null cells (DBS0236862) were obtained from the Dictyostelium stock centre [84]. *pi3K(1-2)* null and *pten* null cells in AX2 background were obtained originally from Dr Richard A. Firtel and Dr Peter N. Devreotes, respectively. *iqgC* null cells in AX2 background (DBS0351225) were generated previously [30]. Cells were cultivated at 22°C in cell culture dishes or shaken in suspension at 150 r.p.m. in HL5 medium without glucose (Formedium) supplemented with 18 g l^−1^ of maltose (Sigma), with added 50 µg ml^−1^ ampicillin and 40 µg ml^−1^ streptomycin. Cells transfected with extrachromosomal expression vectors were maintained in the presence of 10 µg ml^−1^ G418. Transfection of the cells by electroporation and selection of the *rab5A* null clones were performed as described in [85].

### 4.3. Generation of *iqgC* and *rab5A* null cells

To generate *iqgC* null cells in the DdB background, wild-type DdB cells were transfected with the pDM1081_iqgC_KO vector. Transfected cells were selected in SorMC buffer with *K. aerogenes* and 100 µg ml^−1^ of hygromycin B (Sigma). Initial screening for disruption of the *iqgC* gene was done by PCR with primers complementary to the hygromycin cassette and *iqgC* gene fragment downstream from the 3′-region of homology. Positive clones were further analyzed by southern blot. gDNA was digested with PacI and hybridized with a probe complementary to the hygromycin resistance cassette (Hyg probe). After detection, the membrane was stripped and rehybridized with the probe complementary to the *iqgC* gene downstream from the 3′-region of homology (R probe). Probes were digoxigenin-labelled by a PCR DIG Probe Synthesis Kit (Roche) and DNA bands after hybridization were visualized using a DIG Luminescent Detection kit (Roche) on chemiluminescence imaging system Alliance Q9 mini (Uvitec). Finally, a western blot with polyclonal anti-IqgC antibody was used to confirm the lack of protein expression. Phenotypes of two independent clones were identical and one clone (IW023) was used in experiments. To generate *rab5A* null cells in the AX2 background, wild-type AX2 cells were transfected with SalI/BamHI cut and dephosphorylated pLPBLP_rab5A_KO vector. Transfected cells were selected in the presence of 10 µg ml^−1^ blasticidin S (Sigma). Disruption of the *rab5A* gene was initially screened by PCR with primers complementary to the blasticidin cassette and the *rab5A* gene fragment downstream from the 3′-region of homology. Positive clones were further analysed by southern blot on gDNA digested by MlyI and hybridized with a probe complementary to the blasticidin S deaminase CDS (Bsr probe). Successful inactivation of the *rab5A* gene (clone IW031) was also confirmed by Sanger sequencing of gDNA over the *rab5A* locus.

### 4.4. Macropinocytosis, phagocytosis and exocytosis assays

To compare the efficiencies of fluid phase uptake and exocytosis between DdB wild-type, *iqgC* null (*ko*), and *iqgC* null cells with rescued expression of IqgC (*rsc*), cells were washed of bacteria with SorMC buffer and transferred to HL5 medium supplemented with 10% FBS 24 h before the experiment to adapt to axenic growth. For macropinocytosis assessment, the kinetics of uptake of the fluorescently labelled medium was determined during 180 min. 2.5 × 10^5^ cells were incubated in HL5 medium supplemented with 10% FBS and 2.5 µg ml^−1^ Dextran Alexa Fluor 647 (MW 10 000, Life Technologies), and cells were collected at each time point. After washing in a medium with added 0.1% sodium azide to prevent exocytosis, fluorescence was measured by flow cytometry (CytoFLEX S, Beckman Coulter) and analysed with CytExpert software. From the fluorescence value at each time point a background (0 min) value was subtracted, and it was normalized relative to the wild-type control at 120 min. Fluid uptake was calculated as the average of at least 5 independent experiments. To measure exocytosis, cells were incubated in the same medium as for fluid uptake assessment for 120 min. After incubation, cells were washed of fluorescent medium and sampled over the course of 240 min. At each time point cells were sampled and washed in a medium with added 0.1% sodium azide, and fluorescence was measured by flow cytometry (CytoFLEX S, Beckman Coulter). Exocytosis for each strain was normalized relative to the 0 min time point value of wild-type cells, and an average was calculated from at least 5 independent experiments. To assess particle uptake, cells were washed of bacteria with HL5 medium and incubated in HL5 medium supplemented with 10% FBS and 1 or 2 µm in diameter FITC labelled beads (Fluorestbrite carboxy YG microspheres, Polyscience) at ratios 1:200 and 1:17, respectively. The kinetics of uptake was determined during 60 min. At each time point cells were washed with a medium containing 0.1% sodium azide to prevent exocytosis and fluorescence was measured using a microplate reader (Spark, Tecan). From the fluorescence value at each time point a background (0 min) value was subtracted, and it was normalized relative to the appropriate wild-type control at 60 min. Particle uptake was calculated as an average of at least 5 independent measurements. The absolute number of beads internalized by cells was determined by flow cytometry.

### 4.5. Yeast two-hybrid assay

Yeast two-hybrid assay was performed using Matchmaker GAL4 Two-Hybrid System 3 (Clontech Laboratories). *S. cerevisiae* strain AH109 was transfected with the appropriate pair of pGBKT7 and pGADT7 plasmids and successful co-transfection was confirmed by growth on selective plates lacking leucine and tryptophan (-LW). Direct protein interactions were assessed on plates lacking leucine, tryptophan, and histidine (-LWH) in the presence of 0.5 mM 3-amino-1,2,4-triazole (3-AT).

### 4.6. Lipid–protein interaction assay

Lipid dot blot assays were performed using PIP Strip and PIP Array membranes (Echelon Biosciences). To examine the binding of IqgC purified from bacteria, assays were performed according to the manufacturer's instructions. To analyse the binding of truncated or full-length mutated variants of IqgC expressed in *D. discoideum* cells, the assay was performed as described in [44]. Briefly, 1 × 10^7^ cells were washed in Sørensen phosphate buffer, lysed in 600 µl RIPA buffer (Sigma), and incubated on ice for 45 min. PIP Strip membranes were blocked in 3% fatty acid-free bovine serum albumin (Sigma) in PBS-T (8 g l^−1^ NaCl, 0.2 g l^−1^ KCl, 1.42 g l^−1^ Na_2_HPO_4_, 0.245 g l^−1^ KH_2_PO_4_, 0.1% Tween 20, pH 7.2). After centrifugation at 16.000×*g* for 20 min at 4°C lysates were diluted in PBS-T and incubated with membranes for 1 h at room temperature. After washing, membranes were incubated with polyclonal anti-IqgC antibody. In experiments where no binding was detected, a drop of purified IqgC at the bottom of PIP Strip was used as a positive control.

### 4.7. Purification of recombinant proteins and GST pull-down assays

GST-tagged RasG(wt), Rab5A(wt), IqgC_FL, IqgC_GRD, and IqgC_RGCt proteins were expressed in *E. coli* strain Rosetta 2 after induction with 0.75 mM isopropyl-β-D-thiogalactoside (IPTG) at 21°C, overnight. The expression of GST-EEA1^(1–209)^ was induced with 0.3 mM IPTG at 16°C, overnight. Recombinant proteins were affinity purified from bacterial lysates using glutathione-agarose (Thermo Scientific) according to the manufacturer's instructions. Expressions of Rab5A-His variants (wt, Q68L, and S23N) in *E. coli* strain Rosetta 2 were induced with 0.8 mM IPTG at 21°C for 3 h. His-tagged proteins were purified from bacterial lysates by affinity chromatography on a HisTrap HP column (GE Healthcare Europe GmbH) using the chromatography system Äkta FPLC (GE Healthcare Europe GmbH) according to the protocol published in [86]. Rab5A(wt) and (Q68L) were additionally purified by gel-filtration on a HiLoad 16/600 Superdex 200 pg column (GE Healthcare Europe GmbH) using the chromatography system Äkta pure (Cytiva, Global Life Sciences Solutions). Fractions containing Rab5A protein were pooled, dialysed against glycerol (1× PBS, pH 7.3, 1 mM MgCl_2_, 50% glycerol), and stored at −20°C.

To test the direct interaction of IqgC and Rab5A, previously purified IqgC [30] was added to the affinity purified GST-Rab5A(wt) bound to glutathione-agarose and binding of IqgC was confirmed by immunoblotting with a polyclonal anti-IqgC antibody [30]. In addition, previously purified GST-IqgC [30] bound to glutathione-agarose was incubated with purified His-tagged Rab5A variants, and interactions were analysed by immunoblotting with a monoclonal anti-His_6_ antibody (Roche, 11922416001). To examine the Rab5A binding to the RasGAP and RGCt domains of IqgC, GST-IqgC_GRD and GST-IqgC_RGCt bound to glutathione-agarose were incubated with purified Rab5A variants, and again, immunoblotting with a monoclonal anti-His_6_ antibody was performed to assess the interaction. The composition of the buffer used in pull-down assays was as follows: 50 mM Tris, pH 8, 1 mM EGTA, 40 mM NaCl, 1 mM DTT, 1 mM PMSF, 6.6 mM benzamidine, 0.5% NP40. Corresponding GST pull-downs served as a negative control. To compare the levels of active YFP-Rab5A in AX2 wild-type and *iqgC* null cells, GST-EEA1^(1–209)^ bound to glutathione-agarose was used to pull-down active YFP-Rab5A from cell lysates prepared in the lysis buffer (50 mM Tris, pH 8, 1 mM EGTA, 40 mM NaCl, 1 mM DTT, 1 mM PMSF, 6.6 mM benzamidine, 0.5% n-octylpolyoxyethylene, cOmplete EDTA-free protease inhibitors). Bound YFP-Rab5A was immunoblotted with a polyclonal anti-GFP antibody (Abcam, ab290). The relative binding of active Rab5A to EEA1^(1–209)^ in wild-type and *iqgC* null cells was determined by densitometric analysis of YFP-Rab5A expression levels in the respective cell lines. To examine the binding of Rab5A mutant variants to IqgC, GST-IqgC bound to glutathione-agarose was used to pull-down YFP-Rab5A mutant proteins from the lysates of wild-type cells transfected with the corresponding construct. Bound proteins were immunoblotted with a monoclonal anti-GFP antibody (a kind gift from Dr Jan Faix). The relative binding of Rab5A mutant to IqgC was determined by densitometric analysis of YFP-Rab5A proteins' expression levels in cell lysates.

### 4.8. GAP assay

GAP activity of IqgC was determined using the GTPase-Glo Assay kit (Promega) according to the manufacturer's protocol. To test the IqgC GAP activity toward Rab5A, the GTPase activity of 1 µM Rab5A(wt) was determined in the absence and presence of purified IqgC (doubling dilutions from 4.3 to 0.27 µM were used) in a GAP buffer containing 10 µM GTP in 384-well plates. After 2 h of incubation, the GTP that remained in the solution was converted to ATP by nucleoside-diphosphate kinase. Generated ATP was used in a luciferase reaction to produce light. Hence, the higher the GTP hydrolysis, the lower the luminescence output, which was measured on a multimode plate reader (Infinite 200, Tecan). To evaluate the effect of Rab5A on the RasGAP activity of IqgC toward H-Ras, the GAP assay with 1 µM H-Ras (Calbiochem) and 2.2 µM IqgC was performed in the absence and presence of 1 µM Rab5A(Q68L). To assess the effect of Rab5A on the IqgC GAP activity toward *D. discoideum* RasG, the GAP assay with RasG(wt) immobilized on glutathione-agarose and 0.14 µM IqgC was performed in the absence and presence of 1 µM and 2 µM Rab5A(Q68L) in 96-well plates.

### 4.9. Confocal microscopy and image analysis

Localization studies were carried out on a laser scanning confocal microscope (Leica TCS SP8 X), equipped with an HC PL APO CS2 63×/1.4 oil objective, a 405 nm diode laser, and a supercontinuum excitation laser (Leica Microsystems). For imaging macropinosome formation, cells were prepared in HL5 medium with, or without, added 2 mg ml^−1^ TRITC-dextran (MW 65 000–85 000, Sigma). For imaging phagocytosis, cells in the HL5 medium were mixed with TRITC-labelled yeast particles. For imaging colocalization of golvesin-GFP and mRFP-Rab5A, AX2 cells expressing both proteins were fixed with cold methanol (5 min at −20°C) and stained with DAPI. The excitation wavelengths and detection ranges used for imaging were 405 nm and 430–520 nm for DAPI, 488 nm and 498–550 nm for GFP, 511 nm and 520–565 nm for YFP, 565 nm and 575–650 nm for TRITC, 585 nm and 592–650 nm for mRFP. To track YFP-Rab5A on closed macropinosomes *xyt* imaging at the frequency of 2.58 s per frame was performed. At each time point the intensity of the YFP-Rab5A signal was sampled at several locations along the membrane of the macropinosome (ImageJ, NIH). Value for each time point was obtained by averaging the values from all sampled locations. The measurement began at the moment of macropinosome closure (t0) and extended roughly until the intensity reached its peak. To enable effective comparison and analysis of the resulting intensity over time series, all values were normalized by dividing them with the intensity at t0.

The localization of YFP-IqgC in *pi3K(1-2)* null and *pten* null cells, and colocalizations of YFP-IqgC with TAPP1-mRFP and mRFP-2xFYVE in AX2 cells were monitored on a Zeiss 880 inverted microscope equipped with a 63×/1.4 oil-immersion objective. To determine the temporal evolution of the fluorescent intensities during macropinosome formation and maturation, the intensity of the corresponding signal was measured at multiple locations across the macropinosome's membrane at various time points. Each time point's value was calculated by averaging the readings from all the sampled locations. The resulting values, spanning the time series, were then normalized with respect to the cytosolic background intensity.

To analyse the macropinosome size in DdB cells, and retention of IqgC and active Ras probe on the closed macropinosomes in AX2 cells, imaging was performed on a spinning disc confocal microscope (Dragonfly, Andor, Oxford Instruments) using 100×/1.45 oil objective (Nikon) and Sona 4.2B-6 (sCMOS) camera (Andor, Oxford Instruments). The excitation wavelengths 488 and 514 nm, and detection band pass filters 521/38 and 571/78 nm were used for imaging GFP and YFP, respectively. To determine the macropinosome size, DdB wild-type, *iqgC* null and *iqgC* null cells with rescued IqgC expression, expressing GFP-mRaf1_RBD were imaged in *xyzt* mode at an average speed of 1.8 s per stack, acquiring on the average 8 z-sections per stack. To determine retention times of IqgC and active Ras probe in AX2 cells, wild-type, Rab5A(wt)-overexpressing, Rab5A(Y71A)-overexpressing (only for IqgC retention), and *rab5A* null cells, expressing YFP-IqgC or GFP-mRaf1_RBD were imaged in *xyzt* mode under the same conditions. Macropinosome size was determined by measuring the length of the major axis upon macropinosome closure using ImageJ (NIH). The retention times of IqgC and active Ras probe were measured from the macropinosome closure (t0) until the probe was no longer visible on the macropinosome.

### 4.10. Statistics

An independent two-sample *t*-test, assuming unequal variances between the two datasets, was performed for statistical comparison of data in figure 1*a*,*b*,*g*,*h* between wild-type and knock-out cells. For comparing macropinosome sizes and retention times statistical analyses were performed using the Kruskal–Wallis test (function kruskalwallis) in MATLAB software. Statistical analysis for the GAP assay was performed using one-way ANOVA in MATLAB software (function anova1). The *post hoc* Tukey–Kramer test was used for pairwise comparison. The significance level was set to 5% for all analyses.

## Data Availability

Supplementary material is available online [87].
